# The *GATA1*-HS2 Enhancer Allows Persistent and Position-Independent Expression of a β-globin Transgene

**DOI:** 10.1371/journal.pone.0027955

**Published:** 2011-12-02

**Authors:** Annarita Miccio, Valentina Poletti, Francesca Tiboni, Claudia Rossi, Antonella Antonelli, Fulvio Mavilio, Giuliana Ferrari

**Affiliations:** 1 H. San Raffaele-Telethon Institute for Gene Therapy (HSR-TIGET), Istituto Scientifico H. San Raffaele, Milan, Italy; 2 Department of Biomedical Sciences, University of Modena and Reggio Emilia, Modena, Italy; 3 Laboratory of Gene Expression, Istituto Scientifico H. San Raffaele, Milan, Italy; 4 University Vita-Salute San Raffaele, Milan, Italy; Kanazawa University, Japan

## Abstract

Gene therapy of genetic diseases requires persistent and position-independent expression of a therapeutic transgene. Transcriptional enhancers binding chromatin-remodeling and modifying complexes may play a role in shielding transgenes from repressive chromatin effects. We tested the activity of the HS2 enhancer of the *GATA1* gene in protecting the expression of a β-globin minigene delivered by a lentiviral vector in hematopoietic stem/progenitor cells. Gene expression from proviruses carrying *GATA1*-HS2 in both LTRs was persistent and resistant to silencing at most integration sites in the *in vivo* progeny of human hematopoietic progenitors and murine long-term repopulating stem cells. The *GATA1-*HS2-modified vector allowed correction of murine β-thalassemia at low copy number without inducing clonal selection of erythroblastic progenitors. Chromatin immunoprecipitation studies showed that GATA1 and the CBP acetyltransferase bind to *GATA1*-HS2, significantly increasing CBP-specific histone acetylations at the LTRs and β-globin promoter. Recruitment of CBP by the LTRs thus establishes an open chromatin domain encompassing the entire provirus, and increases the therapeutic efficacy of β-globin gene transfer by reducing expression variegation and epigenetic silencing.

## Introduction

β-thalassemias are a group of autosomal recessive disorders characterized by reduced or absent synthesis of hemoglobin β-chains. Allogeneic bone marrow (BM) transplantation from HLA-matched related donors provides a definitive cure for this disease [1]. Gene therapy, i.e., transplantation of autologous, genetically corrected hematopoietic stem cells (HSCs), is a potential alternative for patients lacking a compatible donor. Gene therapy of β-thalassemia requires efficient transfer and appropriately regulated expression of a β-globin transgene. Lentiviral vectors (LV) carrying a human β-globin gene under the control of its minimal promoter and elements of the ß-globin locus-control region (LCR) transduce repopulating HSCs and allow long-term correction of the murine and human β-thalassemia phenotype in pre-clinical models [2], [3], [4], [5], [6]. Limited therapeutic efficacy has been recently observed in one patient treated with transduced CD34^+^ human hematopoietic stem/progenitor cells (HPCs) [7].

A significant limitation of existing β-globin vectors is variegation and epigenetic silencing of transgene expression, which the activity of the β-globin LCR is not sufficient to overcome [8]. Several attempts have been undertaken to reduce chromatin-mediated silencing, such as the introduction of insulators or scaffold/matrix-attachment regions (S/MARs) in the vector backbone. Insertion of a 1.2-kb insulator derived from a chicken α-globin DNase hypersensitive site (cHS4) in the viral long terminal repeats (LTRs) led to complete correction of the thalassemic phenotype in the erythroblastic progeny of transduced human HPCs *in vitro* and *in vivo* [4], [9]. Insertion of the cHS4 in the LTRs has, however, detrimental effects on the vector titer, which is partially overcome by reducing the insulator size [10], [11]. A 573-bp insulator isolated from the sea urchin arylsulfatase locus (*ArsI*) was reported to protect LV from silencing by maintaining an active chromatin structure in an orientation-dependent manner, though not in all cell types or differentiation stages [12], [13]. Another sea urchin chromatin insulator, *sns5*, was shown to protect a gamma-retroviral vector from the negative influence of chromatin in a mouse erythroid cell line [14]. Addition of S/MARs from the human β-interferon or immunoglobulin heavy chain loci, alone or in combination with insulators and enhancers, reduces silencing and provides position-independent transgene expression in hematopoietic cell lines and primary cells [15], [16], [17]. More recently, a ubiquitously acting chromatin opening element (UCOE) from the human *HNRPA2B1-CBX3* locus has been successfully used to minimize the influences of neighboring chromatin on single-copy transgene expression in hematopoietic cells [18], [19].

An alternative strategy to protect an integrated expression cassette from the effect of the surrounding chromatin is the use of transcriptional enhancers, such as the metallothionein or α-globin HS40 enhancer or the DNase hypersensitive site II (HS2) of the β-globin LCR, which relieve position effects at integration sites in cultured cells [20], [21], [22], [23]. In the class of erythroid-specific enhancers, the *GATA1*-HS2, is a 200-bp element from the gene encoding GATA1 [24], a transcriptional activator that operates as a general switching factor for erythroid development [25]. GATA1 binds to the consensus sequence WGATAR within globin LCRs and regulatory regions of erythroid-specific genes and interacts with CBP, a histone acetyltransferase (HAT) that stimulates GATA1 transcriptional activity [26] by inducing an open chromatin configuration. CBP can directly acetylate GATA1 and, consequently, facilitates GATA1 chromatin occupancy [27], [28]. The HS2 element contains a high affinity, palindromic GATA1 binding element that mediates positive autoregulation of *GATA1* expression [29], [30] and binds the transcription factors CP2 [31] and BCL11A [32].

In this study, we tested the ability of *GATA1*-HS2 in counteracting repressive chromatin effects in the context of LV-delivered transgenes. The element was inserted into the LTRs of a LV expressing a GFP gene under the control of the silencing-prone cytomegalovirus (CMV) promoter or a β-globin minigene under the control of a reduced-size LCR. The activity of these vectors was tested in the progeny of transduced, repopulating murine HSCs and human CD34^+^ cells. We show that the *GATA1*-HS2 is able to increase the probability of long-term transgene expression, and to rescue the thalassemic phenotype at low copy number in a murine model of the disease. Chromatin immunoprecipitation (ChIP) studies showed that GATA1 and CBP bind *GATA1*-HS2-containing LTRs and induce hyperacetylation at specific histone residues throughout the provirus. These results show that an effective approach to shield integrated transgenes from negative chromosomal position effects is represented by the inclusion in LV LTRs of sequences co-targeted by transcription factors and chromatin modifying enzymes.

## Results

### A strategy to achieve persistent and position-independent transgene expression in erythroblastic cells

**Figure 1:**
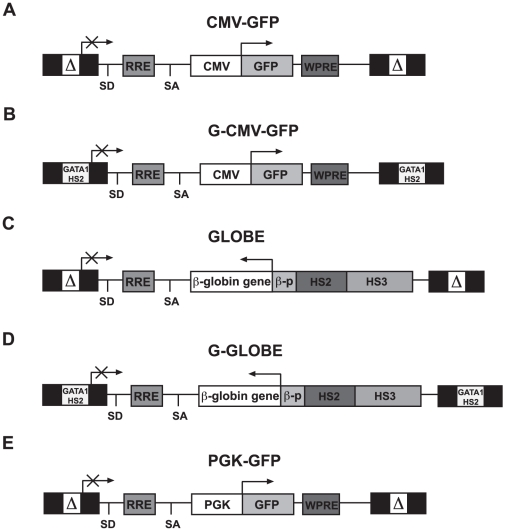
Structure of CMV-GFP, G-CMV-GFP, GLOBE, G-GLOBE and PGK-GFP lentiviral vectors. (A–B) Schematic map of the CMV-GFP and G-CMV-GFP vectors in their proviral form. In both vectors, the CMV promoter drives the expression of a GFP transgene. In the CMV-GFP vector, the *GATA1*-HS2 enhancer was inserted into the self-inactivated LTRs (sin-18 LTRs) carrying 400 bp deletion in HIV U3 region (G sin-18 LTR). The rev-responsive element (RRE), the splice donor site (SD), the splicing acceptor site (SA), the central polypurine tract (cPPT), the woodchuck post-regulatory element (WPRE) are shown. (C) Schematic representation of the β-globin expressing G-GLOBE. The sin-18 LTR, RRE, SD, SA, cPPT, the human β-globin gene, the −265/+50 β-globin promoter (βp) and the human LCR Dnase I hypersensitive sites HS2 and HS3 are shown. (D) Schematic representation of the β-globin expressing G-GLOBE vector. The *GATA1*-HS2 element was inserted into the self-inactivated LTRs (G sin −18 LTR). (E) Structure of the PGK-GFP lentiviral vector. A GFP transgene is under the control of the human phospoglycerate kinase promoter (PGK).

We previously reported that the *GATA1*-HS2 element cloned in transcriptionally active viral LTR drives erythroid-restricted transgene expression in both MLV- and HIV-derived vectors [33], [34]. To test whether *GATA1*-HS2 may positively influence the long-term transgene expression in erythroblastic cells, a previously described [35] LV expressing GFP from the CMV promoter (CMV-GFP, **Figure 1A**) was modified by cloning the 200-bp HS2 element in the 3′ LTR, to obtain the G-CMV-GFP vector **(** **Figure 1B**). The CMV promoter was chosen because of its high susceptibility to silencing *in vitro* and *in vivo* [18]. The activity of the CMV-GFP and G-CMV-GFP vectors was comparatively analyzed following transduction of murine HSCs and human cord blood-derived, CD34^+^ cells.

### The *GATA1*-HS2 element protects the CMV promoter from silencing in the erythroid progeny of murine HSCs

**Figure 2:**
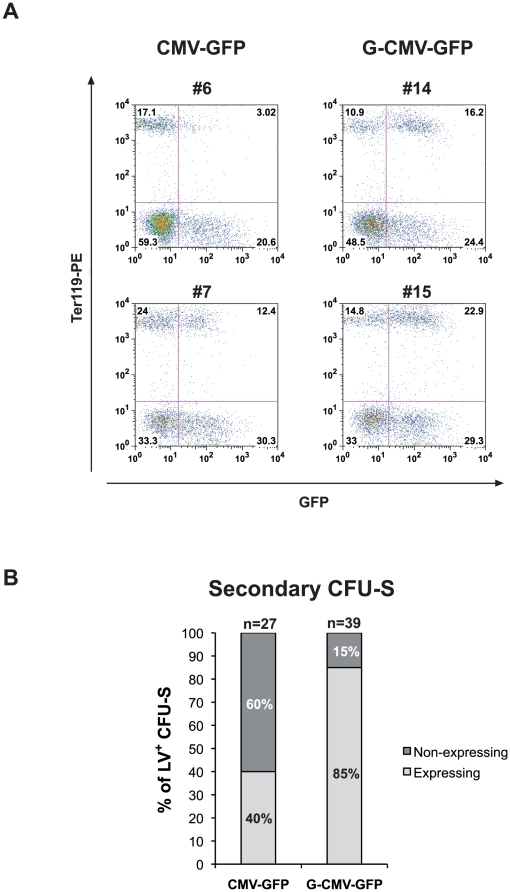
Analysis of transgene expression in the progeny of murine HSCs transduced with CMV-GFP and G-CMV-GFP. (A) Representative FACS analysis of the BM sub-populations of mice transplanted with CMV-GFP-(mice #6 and #7) and G-CMV-GFP-(mice #14 and #15) transduced cells and sacrificed 12 months after BMT. The cells were stained with an antibody that recognizes the erythroid-specific marker Ter119. The presence of *GATA1*-HS2 into the LTRs (G-CMV-GFP vector) is able to sustain transgene expression in the Ter119^+^ BM erythroid compartment. (B) Single erytroid CFU-S were collected from the spleens of secondary recipients 12 days after transplantation with CMV-GFP and G CMV-GFP-containing BM cells (7 and 9 mice, respectively). Donor BM cells are derived from primary transplanted mice which have received CMV-GFP- and G-CMV-GFP-transduced BM cells (3 different donors for each vector). Vector-containing clones carrying a comparable number of proviral copies were analyzed by FACS for transgene expression. The percentages of GFP^+^ (light grey) and GFP^−^(dark grey) vector-positive CFU-S are represented(LV^+^). Transgene expression was observed in 40% of CMV-GFP-containing CFU-S, whereas the presence of *GATA1*-HS2 element into the LTRs was able to sustain GFP expression in 85% of G CMV-GFP-transduced CFU-S. The difference in the proportion of transgene expressing CFU-S between the two groups was statistically significant (P<0.001; Fisher exact test).

BM cells from CD45.1 C57BL/6 (C57BL/6-Ly-5.1) mice were transduced with either the CMV-GFP or the G-CMV-GFP vector and transplanted in lethally-irradiated CD45.2 co-isogenic mice ((C57BL/6; n = 9 and n = 8, respectively). Transduction efficiency, calculated as percentage of GFP^+^ colony forming cells (CFCs) in methylcellulose assay, averaged 75% for both vectors (data not shown). Engraftment of donor cells, as proportion of CD45.1^+^ to total CD45^+^ cells, ranged from 88 to 99% at 12 months after transplantation. BM from recipient animals was analyzed for GFP expression in the erythroid (Ter119^+^) and non-erythroid (Ter119^−^) progeny of transduced, long-term repopulating HSCs. The proportion of Ter119^bright^ cells was comparable in both groups (34±2% and 36±2%, for CMV-GFP and G-CMV-GFP, respectively). GFP^+^ cells were detected at a significantly higher proportion in the Ter119^+^ compartment of mice transplanted with G-CMV-GFP-transduced HSCs, compared to mice transplanted with CMV-GFP-transduced HSCs (56±4% *vs*. 28±8%, P<0.05, **Figure 2A** and **Figure S1A**). The mean fluorescence intensity (MFI) of Ter119^+^/GFP^+^ cells was also significantly higher in mice transplanted with G-CMV-GFP-transduced cells (208±25 *vs*. 96±10, P<0.001, **Figure 2A** and **Figure S1B**). Conversely, the proportion of GFP^+^ cells and the MFI in the Ter119^−^, myeloid and lymphoid compartments were comparable in the two groups (29±11% *vs*. 32±5%: MFI: 372±89 *vs*. 248±23, **Figure 2A**). The average vector copy number (VCN) in total BM cells, analyzed by qPCR, was comparable in both groups (3.5±0.9 for CMV-GFP *vs*. 3.0±0.6 for G-CMV-GFP, P>0.1).

To analyze gene expression in the clonal progeny of long-term HSCs, BM from primary transplanted animals (3 different donors for each vector; **Table S1**) was transplanted in secondary CD45.2 C57BL/6 recipients (n = 8 per group, **Table S1**). A total of 66 12-day colonies derived from spleen colony-forming unit (CFU-S) cells were isolated and analyzed by PCR for the presence of the provirus, and by cytofluorimetry for Ter119 and GFP expression. The proportion of Ter119^bright^ cells was comparable in both groups (data not shown). We observed a >2-fold increase in the percentage of GFP^+^ colonies carrying the G-CMV-GFP vector compared to those carrying the CMV-GFP vector (85% *vs* 40%; P<0.001, **Figure 2B**). The average VCN detected by qPCR analysis was 1.4 and 1.8 for G-CMV-GFP and CMV-GFP colonies, respectively (not shown). These data indicate that the HS2 element significantly increases the probability of transgene expression in the clonal erythroid progeny of murine repopulating HSCs by reducing stable and/or variegating position effect.

### The *GATA1*-HS2 element maintains high level of transgene expression in the erythroid progeny of human HPCs *in vitro* and *in vivo*

**Figure 3:**
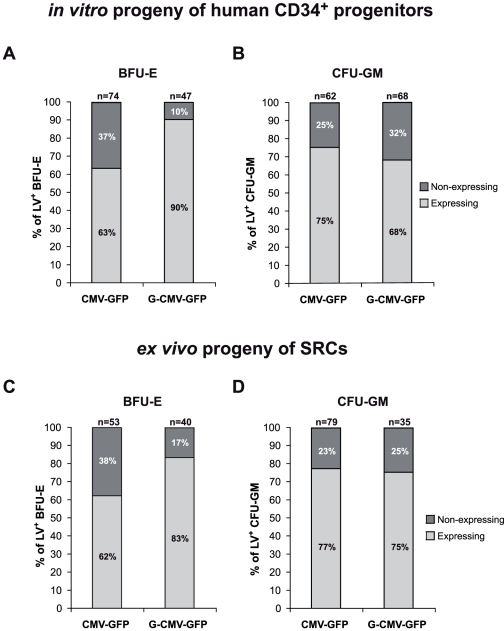
*GATA1*-HS2 is able to sustain transgene expression in the erythroid compartment differentiating from human HPCs. (A–B) BFU-E and CFU-GM derived from CMV-GFP- and G-CMV-GFP-transduced CD34^+^ cells were scored by PCR for vector integration and for GFP expression under fluorescence microscopy. The percentages of GFP^+^ (dark grey) and GFP^−^ (light grey) colonies among the vector-containing BFU-E are represented (for each sample set, n is indicated above the corresponding bar). The proportion of vector-containing BFU-E that do not express the transgene is significantly higher for CMV-GFP compared to the percentage observed in G-CMV-GFP-transduced BFU-E (A; P<0.005 Fisher exact test). (B) No significant difference in the proportions of GFP^+^ CFU-GM was observed between the two groups. (C–D) NOD SCID mice were transplanted with CMV-GFP- and G-CMV-GFP-transduced CB-derived CD34^+^ cells. 8–11 weeks after transplantation mice were sacrificed and BM cells were ex vivo cultured in a medium allowing the growth of human erythroid and myeloid colonies. BFU-E and CFU-GM derived from CMV-GFP- and G-CMV-GFP-transduced SRCs (SCID repopulating cells) were scored by PCR for vector integration. The proportion of vector-positive BFU-E that do not express the transgene is higher for CMV-GFP vector compared to the percentage observed in G-CMV-GFP-transduced BFU-E (P<0.05; Fisher exact test). The proportions of GFP^+^ CFU-GM were similar for both vectors (P>0.1).

We then tested the activity of CMV-GFP and G-CMV-GFP vectors in human cord blood-derived CD34^+^ cells. To analyze transgene expression at clonal level, CMV-GFP- and G-CMV-GFP-transduced CD34^+^ cells were cultured in methylcellulose for 14 days, and single colonies were scored for GFP expression by fluorescence microscopy inspection, and analyzed for the presence of vector sequences by PCR. The vast majority (90%) of cells from BFU-Es (erythroid burst-forming units) carrying the G-CMV-GFP vector expressed the transgene compared to a significantly lower proportion (63%) of those harboring the CMV-GFP vector (P<0.005, **Figure 3A**), while no significant difference was observed in the proportion of GFP^+^ cells from CFU-GM (granulocyte-macrophage colony-forming units) between the two groups (75% *vs*. 68%, P>0.1, **Figure 3B**).

Transduced HPCs were then assayed *in vivo* after transplantation in sub-lethally irradiated NOD-SCID mice (n = 5 and n = 4 for the CMV-GFP and G-CMV-GFP vector). Transduction efficiency ranged from 70 to 80% for both vectors. Eight to 11 weeks after transplantation, the engraftment of SCID-repopulating cells (SRCs) ranged from 8 to 28%, as determined by the proportion of human CD45^+^ cells in the mouse BM. Staining with antibodies against lineage markers (CD19, CD13, and CD34) showed the presence of human lymphoid, myeloid, and undifferentiated progenitors, indicating multilineage differentiation of SRCs in both mouse groups (data not shown). Since human erythroid differentiation occurs at low level in NOD-SCID chimeras, we tested GFP expression in the erythroblastic progeny of transduced SRCs *ex vivo,* in clonal cultures preferentially supporting the growth of human progenitors. BFU-E colonies were scored for both GFP expression and the presence of proviral vectors. Overall, 33 out of 40 (83%) of the G-CMV-GFP-transduced colonies expressed the transgene, compared to 33 out of 53 (62%) in the case of CMV-GFP (P<0.05, **Figure 3C**). The percentage of transduced CFU-GM colonies expressing the transgene was comparable for the two vectors (77% and 75% for CMV-GFP and G-CMV-GFP, respectively; **Figure 3D**). These data show that that *GATA1*-HS2 partially protects a CMV-driven transgene from silencing in the erythroid progeny of human HPCs and SRCs.

### The *GATA1*-HS2 element improves correction of the murine β-thalassemia phenotype by low copy number of a β-globin vector

**Figure 4:**
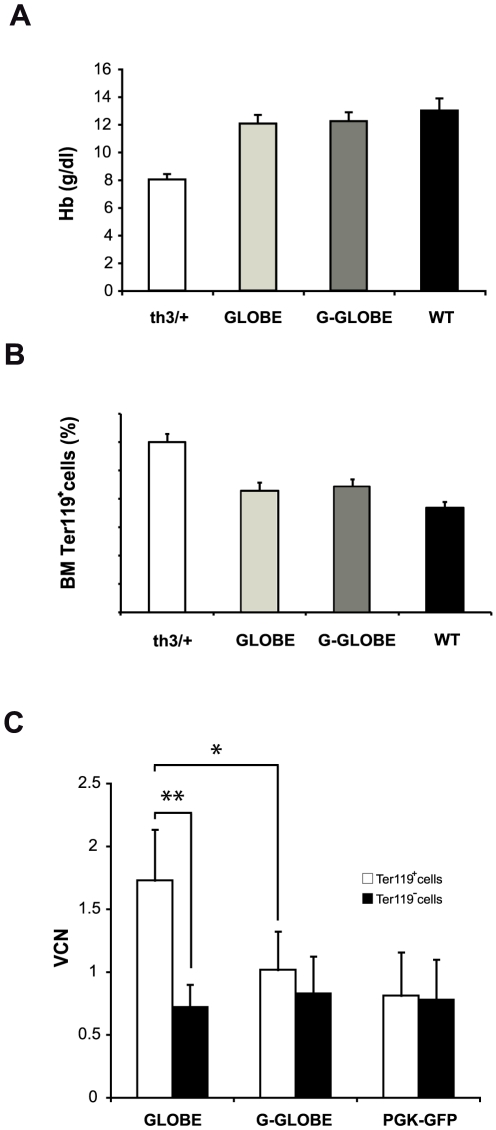
A low G-GLOBE vector copy number is sufficient to correct of the murine thalassemic phenotype. (A) Hb concentration in peripheral blood of mice transplanted with mock-transduced th3/+ (white column; n = 8), normal (WT; black column; n = 9) and GLOBE- (GLOBE; light gray column; n = 8) and G-GLOBE (G-GLOBE; dark gray column; n = 8) transduced BM th3/+ cells. Values represent the mean and SEM for each group of animals. Hb levels were not significantly different in th3/+ mice transplanted with GLOBE- and G-GLOBE-transduced HSCs. (B) Percentages of Ter119^+^ cells in BM of mice transplanted with mock-transduced th3/+ (white column; n = 8), normal (WT; black column; n = 9) and GLOBE- (GLOBE; light gray column; n = 8) and G-GLOBE (G-GLOBE; dark gray column; n = 8) transduced BM th3/+ cells. Values represent the mean and SEM for each group of animals. The proportion of Ter119^+^ erythroid cells were not significantly different in th3/+ mice transplanted with GLOBE- and G-GLOBE-transduced HSCs. (C) Lack of selection of erythroid progenitors carrying multiple copies of G-GLOBE. The Ter119^+^ fraction was sorted from the non-erythroid (Ter119^−^) sub-population of BM cells from mice transplanted with GLOBE-, G-GLOBE- and LV GFP-transduced cells and VCN was determined by qPCR. The proportion of GLOBE-transduced cells in the Ter119^+^ fraction was 2.5 fold higher than that observed in the Ter119^−^ compartment, indicating an *in vivo* selective advantage of highly transduced erythroid progenitors [5] (**P<0.01). In marked contrast, no statistically significant difference was observed for mice receiving G-GLOBE- and GFP-transduced BM cells (P>0.1), thus indicating 1 copy of G-GLOBE per cell is sufficient to produce normal Hb levels (Figure 4A). Interestingly, G GLOBE is able to rescue the thalassemic phenotype with a significantly lower VCN compared to GLOBE (*P<0.05).

We previously showed that the GLOBE LV, expressing a human β-globin gene under the control of the β-globin promoter and a reduced-size LCR (**Figure 1C**) is able to correct the murine β-thalassemia phenotype by providing a selective advantage to genetically corrected erythroblasts [5]. We therefore tested the effect of introducing a copy of *GATA1*-HS2 in both LTRs of the GLOBE vector (G-GLOBE, **Figure 1D**) on the expression of the β-globin gene in a thalassemic mouse model. We used heterozygous C57BL/6-Hbb^th3^ knock-out mice (th3/+) [36], which show a pathophysiology comparable to that of patients with severe ß-thalassemia intermedia, that is chronic anemia, anomalies in RBC size and shape, and ineffective erythropoiesis. We transplanted 16 lethally-irradiated CD45.2, th3/+ mice with BM cells from co-isogenic CD45.1 th3/+ donors, transduced with either GLOBE or G-GLOBE (n = 8 per group). Control mice were transplanted with either wild-type cells (WT control, n = 9) or mock-transduced thalassemic cells (th3/+ control, n = 8). Donor chimerism was >90% at 9–12 months after transplantation in all mice. The extent of phenotypic correction was similar in mice transplanted with G-GLOBE and GLOBE-transduced BM: Hb level, ineffective erythropoiesis (evaluated as percentage of Ter119^+^ cells in BM), hematocrit, RBC and reticulocyte counts were equally improved compared to th3/+ controls (**Figure 4A, 4B** **and Figure S2**). Likewise, expression of human β-globin, as analyzed by FACS analysis and HPLC, was comparable in the two groups (**Table S2**). However, the average VCN in the G-GLOBE-transduced, Ter119^+^ cells was significantly lower than in GLOBE-transduced erythroblasts (1.02±0.36 *vs*. 1.73±0.40, P<0.05), while no difference was observed in the Ter119^−^, myeloid population between the two groups (0.70±0.20 *vs*. 0.83±0.30) (**Figure 4C**). No significant difference in the VCN of Ter119^+^ *vs.* Ter119^−^ cells was observed in th3/+ mice transplanted with HSCs transduced with a GFP-expressing vector (0.81±0.34 *vs*. 0.78±0.32; P>0.1, **Figure 1E** **and** **Figure 4C**). These results indicate that G-GLOBE is able to correct the murine thalassemia at an average VCN of 1 without inducing selection of erythroblasts harboring a higher number of proviruses, as observed for the GLOBE vector.

### The *GATA1*-HS2 element prevents silencing of a β-globin transgene in the progeny of long-term repopulating HSCs

**Figure 5:**
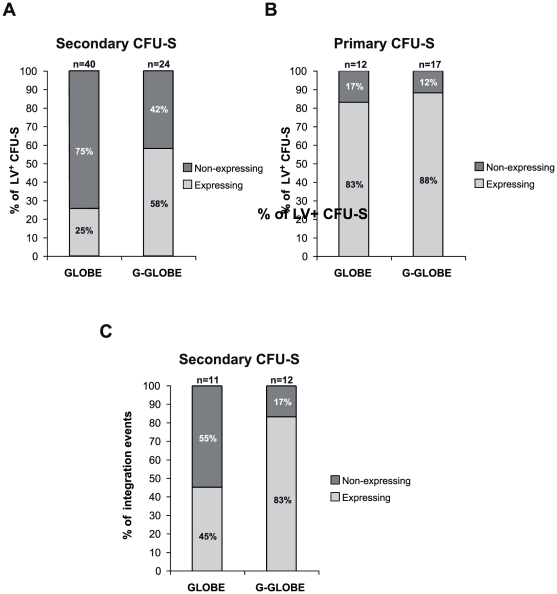
Long-term human β-globin expression in the erythroid progeny of G-GLOBE-transduced HSCs. (A) Single erythroid CFU-S were collected from the spleens of secondary recipients 12 days after transplantation with GLOBE- and G-GLOBE-containing BM cells (16 mice per group). Donor cells are derived from 4 different primary recipients for each vector. LV-containing clones (40 and 24 for GLOBE and G-GLOBE, respectively) carrying single integrants, were analyzed by FACS for β-globin expression. The percentages of β-globin^+^ (light grey) and β-globin^−^(dark grey) LV-containing CFU-S are represented. Transgene expression was observed only in 25% of GLOBE-containing CFU-S, whereas the presence of *GATA1*-HS2 element into the LTRs was able to sustain human β-globin expression in 58% of G-GLOBE-transduced CFU-S. The difference in the proportion of transgene expressing CFU-S between the two groups was statistically significant (*P*<0.005; Fisher exact test). (B) Single erythroid CFU-S were collected from the spleens of mice 12 days after transplantation with GLOBE- and G-GLOBE-transduced BM cells (4 and 7 recipients, respectively. LV-containing clones, carrying on average 1.6 GLOBE and 1.4 G-GLOBE integrants, respectively, were analyzed by FACS for human β-globin expression. Transgene expression was observed in 83% and 88% of CFU-S transduced with GLOBE and G-GLOBE LV (n = 12 and n = 17 for GLOBE and G-GLOBE, respectively). No statistical difference in the proportion of globin expressing CFU-S between the two groups was observed (*P*>0.1; Fisher exact test). (C) GLOBE and G-GLOBE integration sites in secondary CFU-S were cloned by LM-PCR and mapped on the mouse genome. CFU-S harboring individual integration events were analyzed for β-globin gene expression (n = 11 and n = 12 for GLOBE and G-GLOBE, respectively). A 2-fold increase in the proportion of β-globin^+^ CFU-S carrying the G-GLOBE vector was observed compared to the percentage of GLOBE-transduced β-globin expressing colonies.

To test the effect of *GATA1*-HS2 on β-globin transgene expression in the clonal progeny of long-term repopulating HSCs, BM cells harboring 0.3–1 GLOBE and 0.2–1 G-GLOBE average vector copies per cell were harvested from primary transplanted mice (4 donors per group) 12 months after transplantation and injected in wild-type secondary recipients. We mapped 2 to 15 different predominant proviral integration sites in the total BM of mice in the GLOBE group and 3 to 15 in mice in the G-GLOBE group, as analyzed by LM-PCR and sequencing (**Table S1**). Individual colonies from 12-day secondary CFU-S were isolated from recipient mice and analyzed for the presence of the provirus and for β-globin expression. qPCR analysis revealed that all transduced colonies harbored a single proviral copy. Human β-globin synthesis, detected by FACS analysis, was observed in erythroblasts from 14 out of 24 G-GLOBE-containing splenic colonies (58%), compared to 10 out of 40 GLOBE-containing colonies (25%), indicating that *GATA1*-HS2 significantly increases (P<0.005) the probability of transgene expression in the progeny of long-term HSCs (**Figure 5A**). Interestingly, this effect was not apparent in primary colonies from 12-day CFU-S: 10 out of 12 (83%) colonies harboring GLOBE and 15 out of 17 (88%) colonies harboring G-GLOBE expressed human β-globin (P>0.1) (**Figure 5B**). The average VCN was 1.6 for GLOBE-transduced and 1.4 for G-GLOBE-transduced primary colonies.

Proviral integration sites in secondary CFU-S-derived colonies were individually sequenced and mapped by LM-PCR. 5 out of 11 GLOBE and 10 out of 12 G-GLOBE integrations were associated to transgene expression, while the remaining proviruses were always silent (**Table S3 and S4**), showing that a G-GLOBE provirus is less dependent on its genomic position than a GLOBE provirus for transgene expression (45% *vs.* 83% of active proviruses, **Figure 5C**). Indeed, some integrations (3 G-GLOBE and 2 GLOBE), independently identified in different colonies, were variably active (**Table S3 and S4**), indicating that position effect variegation does occur in the progeny of long-term repopulating HSCs. The number of these events was too small to establish whether variegation is reduced by the presence of *GATA1*-HS2 in the LTRs.

### The integration pattern of the G-GLOBE vector is not biased by the presence of the *GATA1*-HS2 element in the LTRs

We previously showed that the introduction of the MLV enhancer in the HIV LTR causes subtle changes in the integration pattern of LV [37]. To test whether the presence of the *GATA1*-HS2 element could bias LV integration, we analyzed the integration site distribution of the CMV-GFP and G-CMV-GFP vectors in the genome of human CD34^+^ HPCs. A total of 438 CMV-GFP and 305 G-CMV-GFP integration sites were mapped by LM-PCR, annotated as TSS-proximal, intragenic and intergenic, and their relative distribution compared to that of 9,974 random control sequences (**Figure S3A)**. Both vectors integrate preferentially into genes (71.2% and 69.5% for CMV-GFP and G-CMV-GFP, respectively, compared to 36.1% in the control sequences, **Figure S3A**) with no preference for TSSs (**Figure S3B**). To correlate vector integration with gene activity, we determined the gene expression profile of CD34^+^ cells at the time of transduction by Affymetrix microarray analysis [6], and analyzed the expression level of the genes targeted by the vectors. As expected [38], both LV targeted preferentially active genes compared to random controls (**Figure S4**). Finally, we correlated vector integration sites and epigenetic modifications obtained from previously published ChIP-sequencing data sets [39]. The ±1-kb regions flanking CMV-GFP and G-CMV-GFP integration sites were equally enriched in PolII binding sites and transcription-associated H3K4me1, H3K36me3, H4K20me1, H3K9me1, and H3K27me1 histone modifications compared to random sequences, with no differences between CMV-GFP and G-CMV-GFP (**Figure S5**). Similarly, H3K4me3, H3K9me3 and H3K27me3 modifications and binding of the H2A.Z histone variant were under-represented around both LV integrations (**Figure S5**). The presence of *GATA1*-HS2 in the LTR has therefore no apparent influence on the selection of integration sites of the LV.

### *GATA1*-HS2 reduces proviral silencing: exploring the mechanism

**Figure 6:**
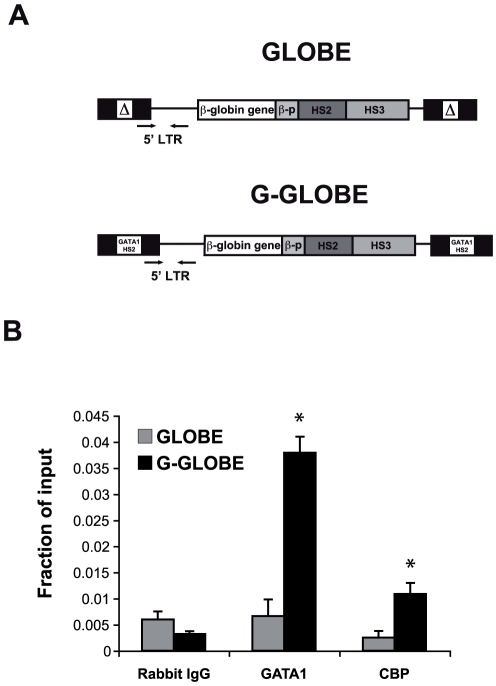
GATA1 and CBP bind to *GATA1*-HS2-containing 5′ LTRs. HEL cells were transduced with GLOBE and G-GLOBE and ChIP assay was performed using antibodies against GATA1 and CBP. (A) Schematic map of GLOBE and G-GLOBE vectors. Arrows indicate the primer pairs used to amplify by PCR the viral 5′ LTR. (B) Chromatin was immunoprecipitated with antibodies against GATA1 and CBP and isotype-matched control antibodies (Rabbit IgG). GATA1 and CBP binding to the 5′LTR of GLOBE and G-GLOBE vectors was measured by real-time PCR. GATA1 and CBP are detected only at the *GATA1*-HS2-containing 5′LTRs (n = 2). *P<0.05 by Student *t* test. Error bars represent SEM.

GATA1 regulates all known erythroid-specific genes, often through the association with the CBP histone acetyltransferase [40]. To investigate whether GATA1 binds to the HS2-containing LTRs and recruits CBP, ChIP experiments were carried out in GATA1-expressing HEL cells transduced with either GLOBE or G-GLOBE LV, using antibodies against GATA1 and CBP. PCR primer pairs were used to amplify sequences corresponding to the 5′ LTR by qPCR analysis (**Figures 6A**). As expected, GATA1 was highly enriched at *GATA1*-HS2-containing LTRs (**Figures 6B**). Accordingly, CBP showed occupancy above background only in G-GLOBE-transduced cells. GATA1 and CBP occupancy at the endogenous β-globin LCR HS4 element, chosen as an internal control, was similar in GLOBE and G-GLOBE-transduced cells (**Figure S6**).

**Figure 7:**
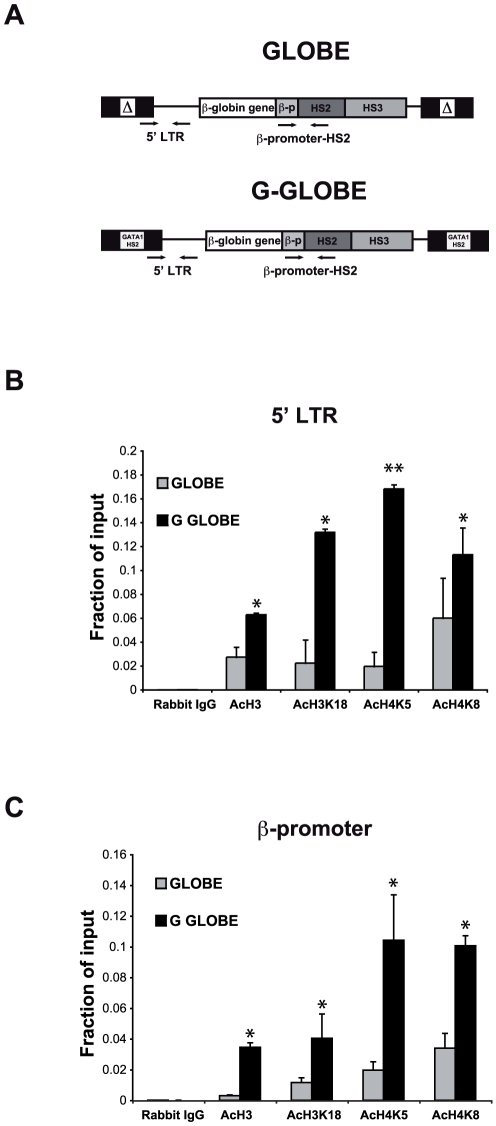
Increased CBP-mediated histone acetylation levels at *GATA1*-HS2-containing LV. ChIP assay was carried out in GLOBE- and G-GLOBE-transduced HEL cells using antibodies against pan-acetylated histone H3, acetylated histone H3K18, acetylated histone H4K5, and acetylated histone H4K8. (A) Map of GLOBE and G-GLOBE proviruses Arrows indicate the primer pairs used to amplify the 5′LTR and the β-globin promoter-HS2 region. The acetylation levels are significantly higher at the 5′ LTRs (B) and at the β-globin promoter region (C) of *GATA1*-HS2-containing LV compared to the GLOBE vector. (n = 4). *P<0.05; **P<0.01 by Student *t* test. Error bars represent SEM.

We then measured histone acetylation levels at different regions in the GLOBE and G-GLOBE proviruses in transduced HEL cells. Primers specific for the 5′LTR and the β-globin promoter (β-promoter-HS2) were used to amplify ChIP products by qPCR (**Figures 7A**). Chromatin was immunoprecipitated using antibodies against pan-acetylated H3 and specific acetylated lysines of H3 and H4 (H3K18, H4K5 and H4K8), which are targets of CBP activity. The G-GLOBE provirus exhibited higher H3 and H4 acetylation levels at the 5′LTR compared to the GLOBE provirus (**Figure 7B**). This difference is maintained at the β-globin promoter region (**Figure 7C**), suggesting the spreading of acetylated histone marks along the chromatin fiber. Histone acetylation levels of the control, transcriptionally active Aldolase A gene were comparable in both GLOBE- and G-GLOBE- transduced cells (**Figure S7**). Concomitantly, the H3K4 trimethylation (H3K4me3), typical of active promoters, tended to be higher at the 5′ LTR of G-GLOBE compared to GLOBE, whereas two heterochromatin marks (H3K9me3 and H3K27me3) were reduced (**Figure S8A and S8B**). H3K4me1, which marks enhancer regions [41], was increased at the *GATA1*-HS2-containing 5′LTR (**Figure S8B**). Overall, these data indicate that GATA1, together with CBP, maintains an open chromatin structure by increasing the histone acetylation levels in the chromatin domain delimited by the *GATA1*-HS2-modified LTRs.

## Discussion

Gene therapy of thalassemia requires vectors expressing high, persistent and regulated levels of β-globin. The use of β-globin promoters, enhancers, and reduced versions of the LCR allows the expression of potentially therapeutic levels of β-globin in the context of LV, as demonstrated in human thalassemic erythroblasts in vitro [6], [9] and in murine models of thalassemia in vivo [2], [5], [42], [43]. LV integrate in different sites in the genome, and although they prefer open and transcribed chromatin regions [44], their expression is susceptible to chromosomal position effects leading to transgene silencing or variegated expression [2], [8], [45]. The reduced versions of the β-globin LCR are apparently insufficient to overcome position-dependent variability of gene expression in erythroblasts. Chromatin insulators, such as the cHS4 element, reduce only partially positional effects, while affecting both titer and genetic stability of the vectors [7], [10], [11]. The outcome of the only patient treated so far with a β-globin LV indicates a limited contribution of the vector-derived protein in the presence of a low number of engrafted transduced HPCs [7]. Low transduction efficiency and genetic instability play a significant role in this still sub-optimal vector performance.

Transcriptional enhancers can suppress negative chromatin influences on transgene expression [21],[22], and might act more consistently and in a larger variety of chromatin contexts compared to conventional insulators or barrier elements. Enhancers may also recruit a promoter to a nuclear compartment in which transcription is stably heritable through cell generations [22]. GATA1 is a master transcriptional activator in erythroid development, binding to regulatory elements of most erythroid-specific genes, including the globin enhancers and LCRs. GATA1 recruits CBP [26], which induces open chromatin conformations by acetylating histones at specific residues. The *GATA1*-HS2 element contains a high-affinity, palindromic GATA1-binding element, and binds the transcription factors CP2 [31] and BCL11A [32]. We previously showed that *GATA1*-HS2 acts as an efficient, erythroid-specific enhancer in the context of both retroviral and lentiviral vectors [33], [34]. In this study, we probed the activity of *GATA1*-HS2 in reducing silencing and position-dependent variability of transgene expression in the context of self-inactivating LV in which *GATA1*-HS2 was inserted in both LTRs. The modification had no influence on the target site selection of the vector, which maintained the typical lentiviral preferences for active genes, enriched in H3K36me3, H4K20me1, H3K9me1, and H3K27me1 histone modifications. Nevertheless, *GATA1*-HS2-flanked transgenes showed more persistent and position-independent expression characteristics, indicating an active role of the element in maintaining a permissive chromatin structure in the target cells.

Differentiation of HSCs into erythroblasts is marked by a gradual increase in chromatin condensation, which is associated with gene silencing at most loci. We therefore tested whether *GATA1*-HS2 could prevent inactivation during erythroid differentiation of transgenes delivered to HSCs and HPCs by a LV. Introduction of *GATA1*-HS2 in the viral LTRs effectively protects from silencing an expression cassette driven by a silencing-prone CMV promoter in erythroblasts differentiating from transduced murine HSCs and human CD34^+^ HPCs *in vitro* and in transplantation assays *in vivo*. The positive effect on transgene expression was not observed in the non-erythroblastic lineages, indicating that the presence of the erythroid-specific GATA1 transcription factor is essential for the activity of the *GATA1*-HS2 element. GATA1 is expressed also in megakaryocytes, eosinophils, and mast cells, which cannot be detected at high frequency in fresh BM cells. *In vitro* cell culture experiments will be necessary to unravel the potential GATA1-HS2 anti-silencing effect in these lineages.

We then tested the activity of *GATA1*-HS2 in the LTRs of the GLOBE LV, containing a human β-globin gene under the control of its promoter and a minimal version (HS2+HS3) of the β-globin LCR [5]. The GLOBE vector is able to correct thalassemia in a murine pre-clinical model of gene therapy [5] and in the erythroblastic progeny of human thalassemic HPCs [6]. The therapeutic efficacy is achieved even at low dose of transduced HSCs by *in vivo* selection of transgene-expressing erythroblastic cells, possibly overcoming the negative influence of chromatin surrounding the LV proviruses. We observed that silencing of the β-globin transgene in the erythroid progeny of human HPCs and murine HSCs transduced with the G-GLOBE vector was significantly reduced compared to cells transduced with the GLOBE vector. As a consequence, the G-GLOBE vector allowed correction of murine thalassemia at a lower copy number and without inducing the *in vivo* selection observed with the GLOBE vector [5]. Therefore, reducing position-dependent variability of gene expression increases the vector's therapeutic potential, providing disease correction at lower vector copy number. Remarkably, the LTR modification does not impair the viral titer, probably because of the limited size of the *GATA1*-HS2 (2.0×10^9^ *vs*. 1.4×10^9^ TU/ml for a typical preparation of GLOBE and G-GLOBE, respectively).

The major difference between the two vectors was observed in the progeny of long-term repopulating HSCs, assayed as CFU-S in secondarily transplanted mice, indicating that the *GATA1*-HS2 provides a more effective long-term protection from silencing compared to to the LCR alone. However, position effect variegation, resulting in expression or silencing in different colonies from CFU-S harboring the same provirus, was observed for both vectors, indicating that *GATA1*-HS2 increases the probability of expression but is unable to completely prevent silencing in the long-term progeny of transduced HSCs.

In order to understand the molecular basis of the protective effect of *GATA1*-HS2, we carried out ChIP studies on erythroblastic cells transduced with the GLOBE or G-GLOBE vectors. A typical function of enhancers is to prevent the formation of repressive chromatin structure by controlling histone acetylation and methylation around promoters. Binding of GATA1 to erythroid-specific enhancers during development or differentiation is a key factor in the initiation and maintenance of active chromatin structures [46]. The interaction of GATA1 with CBP/p300 suggests at least one mechanism by which HATs might be brought to specific sites [26],[47]. By modifying chromatin-bound histones or GATA1 itself [28], acetylases enhance transcriptional activity of erythroid-specific loci [27]. In this context, GATA1 is more an “architectural” factor than a simple transcriptional activator. The essential role of GATA1 and CBP for the formation of an erythroid-specific acetylation pattern that is permissive for high levels of gene expression has been reported at the LCR and the murine ß-major globin gene promoter [40], [48]. Our study shows that binding of GATA1 and CBP to the *GATA1*-HS2 element correlates with increased CBP-mediated histone acetylation (H3K18, H4K5, and H4K8) at the level of the LTRs and the internal promoter. Although GATA1 and CBP bind also the LCR elements and the β-globin promoter within the vector, the presence of the *GATA1*-HS2 element at both sides of the transgene apparently increases CBP-mediated histone acetylation and spreads the acetylated histone domain to a chromatin region that encompasses the entire provirus. This might create a more stable, inheritable active chromatin configuration that renders the integrated transgene resistant to silencing in a large proportion of integration sites.

Our results suggest that GATA1 establishes an active epigenetic state across transgenes flanked by *GATA1*-HS2 elements in the context of LV. Therefore, this vector design represents a way to shield integrated transgenes from negative chromosomal position effects, improving the therapeutic potential of LV-mediated gene therapy approaches. Further studies addressing the impact of this lineage-specific enhancer on cellular transcriptional activity are necessary to assess the risk-benefit balance for potential clinical use.

## Materials and Methods

### Lentiviral vectors

The CMV-GFP lentiviral vector was previously described [35]. To construct the G-CMV-GFP vector, an *Eco*RI/*Sac*I fragment containing part of the 3′ LTR of the lentiviral vector pHR2 [49] was subcloned into the *Pvu*II site of the pUC19 plasmid. The plasmid was digested with *Eco*RV and *Pvu*II, in order to generate a −418 to −18 deletion (where +1 is the transcription start site) in the U3 region of the LTR, and ligated with a 200-bp *Bam*H1 human genomic fragment (−856 to −655) containing the *GATA1*-HS2 [24] to obtain a chimerical self inactivating (SIN) LTR. The *Eco*RI-*Sfi*I fragment containing the modified 3′ LTR was then introduced into the *Eco*RI-*Sfi*I sites of the CMV-GFP backbone [50], to obtain the G-CMV-GFP vector. To generate the G-GLOBE vector, the *Eco*RI-*Sfi*I HS2 SIN LTR fragment was introduced into the *Spe*I-*Sfi*I sites of the GLOBE vector backbone. Viral vector stocks were prepared by transient co-transfection of HEK293T cells using a three-plasmid system as previously described [5]. HEK293T cells were kindly provided by Luigi Naldini.

### Transduction of stable cell lines

HEL (human erythroleukemia) cell line was grown in RPMI 1640 supplemented with 10% FBS, 100 U/ml penicillin/streptomycin and 2 mM L-glutamine, and transduced with viral stocks at a multiplicity of infection (MOI) of 20 in the presence of 8 µg/ml polybrene. HEL cells were purchased by ECACC (European Collection of Cell Cultures).

### Transduction and transplantation of murine HSCs and human HPCs

BM cells were harvested from C57BL/6-Ly-5.1 mice (B/6.SJL-CD45^a^-Pep^3b^, Jackson Laboratories), infected with CMV-GFP and G-CMV-GFP vectors, and injected into 8-wk-old C57BL/6 mice (Charles River) as previously described [33]. BM cells from C57BL/6-Hbb^th3^ mice (Jackson Laboratories), were infected and transplanted into 8-wk-old C57BL/6-Ly-5.1 and C57BL/6-Hbb^th3^ mice, as previously described [5]. C57BL/6 or C57BL/6-Ly-5.1 mice were lethally irradiated (950 cGy) and secondarily transplanted with 10^5^ total BM cells from primary transplanted animals. Spleen colony-forming units (CFU-S) were isolated at day 12 from the spleen of 8- to 12-wk-old secondarily transplanted animals. Human CD34^+^ cells were obtained from umbilical cord blood of healthy donors on collection with written informed consent in accordance with the Declaration of Helsinki (TIGET01 protocol). This study was approved by San Raffaele Scientific Institute Ethical Committee. CD34^+^ cells were cultured, transduced and transplanted (2.5×10^5^ cells/mouse) in NOD-SCID mice (Charles River) as previously described [33]. All procedures were approved by the H. San Raffaele Animal Care and Use Committee (IACUC#295).

### Hematological and cell phenotype analysis

Blood samples were collected from transplanted mice and Hb concentration, hematocrit, RBC and reticulocyte counts were measured as described [5]. RBC and CFU-S were stained with PE-conjugated anti-human β-globin antibody as described [5], [51]. Mouse BM and spleen cell phenotyping was carried out using PE-conjugated anti-mouse TER-119 antibody. PE-conjugated anti-mouse CD45.1 and PerCP-conjugated anti-mouse CD45.2 antibodies were used to evaluate donor-host chimerism. Human cell surface phenotype and human cell engraftment in transplanted NOD/SCID mice were determined as described [33]. Unbound antibodies were removed by a final wash with PBS 1% FBS and cells were analyzed using a FACScalibur cytofluorimeter (Becton Dickinson, Mountain View, CA). Ter119^+^ BM cells were purified as previously described [5]. Antibodies are listed in the Supplemental Materials and Methods (Text S1).

### DNA analysis

Genomic DNA was isolated with the QIAGEN QIAmp DNA mini kit. GFP-specific primers were used to detect the presence of CMV-GFP and G-CMV-GFP vectors. Primers that anneal to the human HS2-HS3 LCR sequence were used to detect GLOBE and G-GLOBE vectors. Primer sequences are listed in Supplemental Materials and Methods (Text S1). The average vector copy number (VCN) was measured by qPCR as previously described [5].

### Integration site analysis

Integration sites were cloned by linker-mediated PCR (LM-PCR) as previously described [38]. Briefly, genomic DNA was extracted from human HPCs, murine BM cells or CFU-S, digested with *Mse*I and ligated to an *Mse*I double-stranded linker. LM-PCR was performed with nested primers specific for the linker and the 3′ HIV LTR [38]. PCR products were shotgun-cloned into libraries of integration junctions, which were then sequenced to saturation. Sequences were mapped on the human (UCSC Human Genome Project Working Draft, hg18) or mouse genome (UCSC Mouse Genome Project Working Draft, mm9) by the BLAT genome browser. A matched random integration dataset was created for the human genome as described [38]. We annotated all genes having their transcription start site (TSS) within 50 kb from each integration/random site in either directions. Integrations were annotated as TSS-proximal when occurring within ±2.5 kb from a TSS, intragenic when occurring inside a gene >2.5 kb from a TSS, and intergenic in all other cases. In case of multiple transcript variants, we chose the longest isoform.

### ChIP assay

ChIP assay was performed as described [44]. Samples were quantified using real-time SYBR Green PCR and analyzed using an Applied Biosystems 7900HT system. Serial dilution of total input chromatin was used to generate a standard curve for each primer and sample set. Primers sequences and antibodies are listed in Supplemental Materials and Methods (Text S1).

### Statistical analysis

We used a 2-tailed Student's *t* test to determine whether hematological parameters, fractions of inputs and VCN differed between groups. Fisher's exact test was used to assess whether the difference between two proportions was significant. The statistical analyses were performed using GraphPad Prism Version 4.0b (GraphPad Software Inc., La Jolla, CA).

## Supporting Information

Figure S1 **Increased transgene expression in the Ter119^+^ compartment of mice receiving G-CMV-GFP-transduced HSCs.** GFP expression was analyzed by FACS in the erythroid Ter119^+^ fraction of BM from mice transplanted with CMV-GFP- and G-CMV-GFP-transduced HSCs, as described in the legend to Figure 2. Both MFI and percentage of GFP^+^ cells are significantly higher in mice receiving G-CMV-GFP-transduced cells (*P<0.05; ****P<0.001; n = 9 for CMV-GFP and n = 8 for G-CMV-GFP).
(EPS)

Figure S2 **Similar correction of haematological parameters in mice transplanted with GLOBE- and G-GLOBE-transduced BM.** RBC count, HCT and percentages of reticulocytes, in peripheral blood of mice transplanted with mock-transduced th3/+ (white column; n = 8), normal (WT; black column; n = 9) and GLOBE- (GLOBE; light gray column; n = 8) and G-GLOBE (G-GLOBE; dark gray column; n = 8) transduced BM th3/+ cells. Values represent the mean and SEM for each group of animals. Haematological parameters were not significantly different in th3/+ mice transplanted with GLOBE- and G-GLOBE-transduced HSCs.
(EPS)

Figure S3 **The presence of HS2 does not affect the lentiviral integration pattern in human CD34^+^ hematopoietic progenitors.** (A) Distribution of CMV-GFP and G-CMV-GFP integration sites in the genome of cord blood-derived CD34^+^ cells. Integrations are defined as intergenic (outside genes), intragenic (inside genes) and TSS-proximal (at a distance of ±2.5 kb from transcriptional start sites, TSSs) as described in the scheme. No statistical differences between the two groups were observed. (B) Distribution of LV integrations sites in human CD34^+^ cells within an interval of 50 kb around the TSSs of known genes. The bars show the percentage of distribution in each 5-kb interval of CMV-GFP and G-CMV-GFP insertions. The red line shows the distribution of computer-generated random insertion sites.
(EPS)

Figure S4 **Correlation between CMV-GFP and G-CMV-GFP integration sites and gene activity.** The bars show the percentage of distribution of expression values from Affymetrix HG-U133A microarrays of CD34^+^ cells at the time of transduction. Target genes are divided in four expression level categories: absent (black), low (below the 25^th^ percentile in a normalized distribution; blue), intermediate (between the 25th and the 75th percentiles; yellow) and high (above the 75th percentile; red). The first bar (Chip CD34^+^) shows the distribution of the genes on the microarray of CD34^+^ cells at the time of transduction, the other three bars represent the expression values of genes at control random sites (Random) or genes targeted by CMV-GFP and G-CMV-GFP integrations. n represents the number of genes.
(EPS)

Figure S5 **Association between histone modifications and CMV-GFP and G-CMV-GFP integrations sites.** Total number of base pairs carrying the indicated histone modification/bound protein on each strand at ±1 kb from each CMV-GFP (green), G-CMV-GFP (blue) and random (grey box) insertion site. The distributions of the entire datasets are represented by box plots. Data were retrieved from ChIP-sequencing experiments performed in the genome of human CD34_+_/CD133_+_ HPCs [39]. Two-sample test statistics among CMV-GFP, G-CMV-GFP and random distributions for each modification indicate that comparisons between CMV-GFP and G-CMV-GFP are not statistically significant (non parametric Mann–Whitney U test, not shown). All comparisons between LV and random distributions are statistically significant (*P<0.05; ****P<0.001; Random: n = 9974; CMV-GFP: n = 438; G-CMV-GFP: n = 305).
(EPS)

Figure S6 **GATA1 and CBP occupancy at the endogenous LCR HS4.** Chromatin from GLOBE- and G-GLOBE-transduced HEL was immunoprecipitated with antibodies against GATA1 and CBP and control rabbit IgG. The endogenous LCR HS4 served as a positive control for GATA1 and CBP binding in both GLOBE- and G-GLOBE-transduced bulk populations (n = 2). Error bars represent SEM.
(EPS)

Figure S7 **Similar acetylation levels at the control AldolaseA gene.** ChIP assay was performed as described in the legend to Figure 7. The levels of histone H3 and H4 acetylation are comparable at the positive control region from Aldolase A gene in both GLOBE- and G-GLOBE-transduced HEL cells (n = 4). The results are averages of 4 independent experiments. Error bars represent SEM.
(EPS)

Figure S8 **Analysis of histone methylation patterns at the 5′LTR and at the β-globin promoter-HS2 region of GLOBE and G-GLOBE vectors.** ChIP assay was carried out in GLOBE- and G-GLOBE-transduced HEL cells using antibodies against mono- and trimethyl-H3K4 (H3K4me1 and H3K4me3) and against trimethyl-H3K9 and trimethyl-H3K27. qPCR was performed to amplify the 5′LTR (A) and the β-globin promoter-HS2 region (B). Bars denote averages of 4 independent experiments. Error bars represent SEM.
(EPS)

Table S1 VCN and number of integrants in primary transplanted mice used as donors for CFU-S assays. The table shows the number of integration sites retrieved from BM of primary transplanted mice, the average vector copy number per BM cell, and the number of CFU-S analyzed for each primary transplanted mouse.
(DOC)

Table S2 Human β-globin expression in mice transplanted with GLOBE- and G GLOBE-transduced BM. The table shows the percentage of human ß-globin positive RBC and the ratio between the areas of HPLC peaks corresponding to human ß and murine α globin chains in mice transplanted with GLOBE- and G GLOBE-transduced BM.
(DOC)

Table S3 List of GLOBE proviral integration sites in secondary CFU-S. The table shows β-globin expression, chromosomal location of integrated provirus, target gene symbol, and RefSeq identifier number in secondary CFU-S. Integrations were annotated as inside or outside (intergenic) known genes (University of California at Santa Cruz annotation).
(DOC)

Table S4 List of G-GLOBE proviral integration sites in secondary CFU-S. The table shows β-globin expression, chromosomal location of integrated provirus, target gene symbol, and RefSeq identifier number in secondary CFU-S. Integrations were annotated as inside or outside (intergenic) known genes (University of California at Santa Cruz annotation).
(DOC)

Text S1 Additional Materials and Methods.
(DOC)
