# Supplementary material for: The GATA1-HS2 Enhancer Allows Persistent and Position-Independent Expression of a β-globin Transgene
Source: PLoS One. 2011 Dec 2;6(12):e27955. doi: 10.1371/journal.pone.0027955 (PMC3229501; doi:10.1371/journal.pone.0027955)
Supplement: Table S1 — VCN and number of integrants in primary transplanted mice used as donors for CFU-S assays. The table shows the number of integration sites retrieved from BM of primary transplanted mice, the average vector copy number per BM cell, and the number of CFU-S analyzed for each primary transplanted mouse. (DOC) [file pone.0027955.s009.doc]

**Table S1. VCN and number of integrants in primary transplanted mice used as donors for CFU-S assays.**

| **# mouse** | **LV** | **# integrations*** | **VCN**** | **# CFU-S***** |
| --- | --- | --- | --- | --- |
| 1 | CMV-GFP | ND | 2.68 | 11 |
| 6 | CMV-GFP | ND | 2.38 | 10 |
| 7 | CMV-GFP | ND | 2.66 | 6 |
| 14 | G-CMV-GFP | ND | 2.84 | 12 |
| 15 | G-CMV-GFP | ND | 2.75 | 13 |
| 16 | G-CMV-GFP | ND | 3.35 | 14 |
| 14 | GLOBE | 15 | 0.33 | 7 |
| 19 | GLOBE | 4 | 1.05 | 4 |
| 4 | GLOBE | 2 | 0.45 | 10 |
| 55 | GLOBE | 13 | 0.71 | 19 |
| 7 | G-GLOBE | 7 | 0.41 | 3 |
| 8 | G-GLOBE | 3 | 0.17 | 2 |
| 11 | G-GLOBE | 14 | 1.13 | 10 |
| 15 | G-GLOBE | 15 | 1.04 | 9 |

* Number of integration sites retrieved from BM of primary transplanted mice, as determined by LM-PCR.

** VCN, the average vector copy number per cell in BM, as determined by qPCR.

***Number of CFU-S analyzed for each primary transplanted mouse.

ND : not determined.
