# Supplementary material for: The GATA1-HS2 Enhancer Allows Persistent and Position-Independent Expression of a β-globin Transgene
Source: PLoS One. 2011 Dec 2;6(12):e27955. doi: 10.1371/journal.pone.0027955 (PMC3229501; doi:10.1371/journal.pone.0027955)
Supplement: Table S2 — Human β-globin expression in mice transplanted with GLOBE- and G GLOBE-transduced BM. The table shows the percentage of human ß-globin positive RBC and the ratio between the areas of HPLC peaks corresponding to human ß and murine α globin chains in mice transplanted with GLOBE- and G GLOBE-transduced BM. (DOC) [file pone.0027955.s010.doc]

**Table S2. Human** β**-globin expression in mice transplanted with GLOBE- and G GLOBE-transduced BM.**

| **# mouse** | **LV** | **%hu**β**^+^rbc §** | **%hu**β**/mα** |
| --- | --- | --- | --- |
| 14 | GLOBE | 64 | ND |
| 19 | GLOBE | 99 | 19 |
| 21 | GLOBE | 95 | 19 |
| 4 | GLOBE | 99 | 23 |
| 6 | GLOBE | 86 | 14 |
| 5 | GLOBE | 99 | 37 |
| 2 | GLOBE | 95 | 30 |
| 55 | GLOBE | 96 | ND |
| 7 | G GLOBE | 99 | ND |
| 8 | G GLOBE | 99 | ND |
| 9 | G GLOBE | 70 | ND |
| 10 | G GLOBE | 97 | ND |
| 11 | G GLOBE | 99 | 24 |
| 12 | G GLOBE | 99 | 32 |
| 13 | G GLOBE | 99 | 35 |
| 15 | G GLOBE | 92 | 20 |

Analyses were performed 6 months after BMT

§percentage of human ß-globin positive (huß ^+^) RBC, as determined by FACS staining.

**= **ratio between the areas of HPLC peaks corresponding to human ß and murine α globin chains (huβ/mα).

ND : not determined.
