# Supplementary material for: The GATA1-HS2 Enhancer Allows Persistent and Position-Independent Expression of a β-globin Transgene
Source: PLoS One. 2011 Dec 2;6(12):e27955. doi: 10.1371/journal.pone.0027955 (PMC3229501; doi:10.1371/journal.pone.0027955)
Supplement: Table S3 — List of GLOBE proviral integration sites in secondary CFU-S. The table shows β-globin expression, chromosomal location of integrated provirus, target gene symbol, and RefSeq identifier number in secondary CFU-S. Integrations were annotated as inside or outside (intergenic) known genes (University of California at Santa Cruz annotation). (DOC) [file pone.0027955.s011.doc]

**Table S3. List of GLOBE proviral integration sites in secondary CFU-S.**

| **CFU-S #** | **donor mouse #** | β**-globin expression** | **chromosomal band** | **Target gene** | **Location** | **RefSeq #** |
| --- | --- | --- | --- | --- | --- | --- |
| 10.1 | 55 | negative | chr6qA1 |  | intergenic |  |
| 12.1 | 55 | negative | chr6qA2 |  | intergenic |  |
| 10.6 | 55 | negative | chr9qA2 |  | intergenic |  |
| 10.5 | 55 | negative | chr10qD2 |  | intergenic |  |
| 11.1 | 55 | negative | chr18qE2 |  | intergenic |  |
| 4.3 | 4 | positive | chr3qA1 |  | intergenic |  |
| 11.2 | 4 | negative | chr8qE.1 | Usp10 | intron 1 | NM_009462.1 |
| 11.3 | 4 | negative | chr8qE.1 | Usp10 | intron 1 | NM_009462.1 |
| 2.1 | 4 | positive | chr8qE.1 | Usp10 | intron 1 | NM_009462.1 |
| 4.1 | 4 | positive | chr8qE.1 | Usp10 | intron 1 | NM_009462.1 |
| 4.7 | 4 | positive | chr8qE1 | Usp10 | intron 1 | NM_009462.1 |
| 3.1 | 4 | positive | chrXqA5 | Hs6st2 | intron 2 | NM_001077202.1 |
| 8.2 | 14 | negative | chr6qA1 |  | intergenic |  |
|  |  |  | chr8qB3.1 |  | intergenic |  |
| 5.5 | 14 | negative | chr6qA1 |  | intergenic |  |
|  |  |  | chr8qB3.1 |  | intergenic |  |
| 6.3 | 14 | negative | chr15qA1 | Lmbrd2 | intron 4 | NM_177178.3 |
| 8.1 | 14 | positive | chr15qA1 | Lmbrd2 | intron 4 | NM_177178.3 |
| 6.6 | 14 | negative | chr15qA1 | Lmbrd2 | intron 4 | NM_177178.3 |
| 9.1 | 19 | positive | chr12qC1 |  | intergenic | NM_178714.4 |
| 9.2 | 19 | positive | chr12qC1 |  | intergenic | NM_178714.4 |
| 10.2 | 19 | positive | chr12qC1 |  | intergenic | NM_178714.4 |
| 11.1 | 19 | positive | chr12qC1 |  | intergenic | NM_178714.4 |

Columns indicate, from left to right: CFU-S number, mouse number, β-globin expression, chromosomal location of each integrated provirus, target gene symbol, and RefSeq identifier #. Integrations were annotated as inside or outside (intergenic) known genes (University of California at Santa Cruz annotation).
