# Supplementary material for: The GATA1-HS2 Enhancer Allows Persistent and Position-Independent Expression of a β-globin Transgene
Source: PLoS One. 2011 Dec 2;6(12):e27955. doi: 10.1371/journal.pone.0027955 (PMC3229501; doi:10.1371/journal.pone.0027955)
Supplement: Table S4 — List of G-GLOBE proviral integration sites in secondary CFU-S. The table shows β-globin expression, chromosomal location of integrated provirus, target gene symbol, and RefSeq identifier number in secondary CFU-S. Integrations were annotated as inside or outside (intergenic) known genes (University of California at Santa Cruz annotation). (DOC) [file pone.0027955.s012.doc]

**Table S4. List of G-GLOBE proviral integration sites in secondary CFU-S.**

| **CFU-S #** | **donor mouse #** | **β-globin expression** | **chromosomal band** | **Target gene** | **Location** | **RefSeq #** |
| --- | --- | --- | --- | --- | --- | --- |
| 14.2 | 11 | positive | chr5qG2 | Auts2 | intron 6 | NM_177047.3 |
| 14.3 | 11 | positive | chr5qG2 | Auts2 | intron 6 | NM_177047.3 |
| 15.7 | 11 | positive | chr6qB2.2 | Cntnap2 | intron 1 | NM_001004357.1 |
| 15.6 | 11 | negative | chr18qE2 | Dcc | intron 1 | NM_007831.3 |
| 13.6 | 11 | positive | chr19qA | 1700123I01Rik | intron 2 | NM_001165919.1 |
| 14.1 | 11 | positive | chrXqA5 |  | intergenic | NM_133729.1 |
| 15.8 | 11 | positive | chrXqA5 |  | intergenic | NM_133729.1 |
| 14.4 | 11 | positive | chrXqA5 |  | intergenic | NM_133729.1 |
| 15.2 | 11 | positive | chrXqA5 |  | intergenic | NM_133729.1 |
| 20.3 | 7 | positive | chr6qG1 |  | intergenic |  |
| 20.1 | 7 | positive | chr19qC3 | Nt5c2 | intron 2 | NM_029810.4 |
| 20.2 | 7 | negative | chr19qC3 | Nt5c2 | intron 2 | NM_029810.4 |
| 4.6 | 15 | positive | chr3qA3 | Tnfsf10 | intron 1 | NM_009425.2 |
| 4.5 | 15 | positive | chr14qA2 | Slc4a7 | intron 1 | NM_001033270.2 |
| 4.8 | 15 | negative | chr14qA1 | Slc4a7 | intron 1 | NM_001033270.2 |
| 4.9 | 15 | positive | chr18qE2 | Smad4 | intron 5 | NM_008540.2 |
| 4.7 | 15 | negative | chrXqD |  | intergenic | NM_007674.3 |
| 5.2 | 8 | negative | chr1qF |  | intergenic |  |

Columns indicate, from left to right: CFU-S number, mouse number, β-globin expression, chromosomal location of each integrated provirus, target gene symbol, and RefSeq identifier #. Integrations were annotated as inside or outside (intergenic) known genes (University of California at Santa Cruz annotation).
