# Supplementary material for: The GATA1-HS2 Enhancer Allows Persistent and Position-Independent Expression of a β-globin Transgene
Source: PLoS One. 2011 Dec 2;6(12):e27955. doi: 10.1371/journal.pone.0027955 (PMC3229501; doi:10.1371/journal.pone.0027955)
Supplement: Text S1 — Additional Materials and Methods. (DOC) [file pone.0027955.s013.doc]

**Supplemental Materials and Methods**

**Primers used for PCR (5’ to 3’)**

GFP FOR ATGGTGAGCAAGGGCGAG

GFP REV GTTACTTGTACAGCTCGTC

HS2 FOR GTTGGAGGATACCCATTCTCTATCT

HS3 REV TGGGTCAGTGGTCTCAATGTAGCA

**Primers used for realtime SYBR green ChIP analysis (5’ to 3’)**

5' LTR FOR TAGTGTGTGCCCGTCTGTTG

5' LTR REV CTCTCGCACCCATCTCTCTC

Endogenous LCR HS4 FOR TGCCCTGCCTCTCTACTAGG

Endogenous LCR HS4 REV ATGGAAATGATCAGGCTTGG

**Antibodies used for flow cytometry**

Name Conjugate(s) Clone Source

Ter119 PE TER-119 BD Pharmingen

CD45.1 PE A20 BD Pharmingen

CD45.2 PerCP 30-F11 BD Pharmingen

CD45 PE PD7/26 -2B111 DAKO

anti-human ß-globin PE 37-8 Santa Cruz Biotechnology

**Antibodies used for ChIP**

Name Clone Source

Rabbit IgG polyclonal DBA

GATA1 polyclonal Abcam

KAT3A/ CBP polyclonal Abcam

Histone H3 (acetyl-K14) polyclonal Millipore

Histone H3 (acetyl-K18) polyclonal Abcam

Histone H4 (acetyl-K5) polyclonal Upstate

Histone H4(acetyl-K8) polyclonal Millipore

Histone H3 (monomethyl K4) polyclonal Abcam

Histone H3 (trimethyl K4) polyclonal Millipore

Histone H3 (trimethyl K9) polyclonal Abcam

Histone H3 (trimethyl K27) polyclonal Millipore

**Protein analysis**

Globin polypeptide chain composition was determined by HPLC as described in [1].

**Gene expression profiling**

The expression profile of CD34^+^ cells was determined by Affymetrix microarray analysis as previously described[2]. To correlate retroviral integration and gene activity, expression values were divided into four classes, i.e. absent, low (below the 25^th^ percentile in a normalized distribution), intermediate (between the 25^th^ and the 75^th^ percentile) and high (above the 75^th^ percentile).

# Statistical analyses

For the pairwise comparisons regarding the LV integration distribution analysis, and the correlation between gene activity and LV integration sites, we applied a 2-sample test for equality of proportions with continuity correction. The statistical analyses were performed using the R web 1.03 statistical analysis package.

**Genome-wide epigenetic association studies**

To correlate histone modifications and retroviral integration frequency, a window of 1 kb around each insertion/random site was annotated with a number of histone methylation sites or chromatin bound proteins obtained from publicly available ChIP-seq data in human CD34^+^/CD133^+^ hematopoietic progenitor cells [3].

For each modification/bound protein, we retrieved the read coordinate bed file (<http://dir.nhlbi.nih.gov/papers/lmi/epigenomes/hghscmethylation.aspx>), sorted it by both strand and start position, and merged overlapping regions belonging to the same strand. The merged coordinate file was used to assign a “modification score” to each base pair in the genome, as follows: 0 if no modification/bound protein was found (no intervals contained that base pair); 1 if a base pair was modified on one strand only; 2 for base pairs modified on both strands. For the 1,000 bp upstream and downstream of each insertion/random site we then summed the modification scores, obtaining values ranging from 0 to 4,000. The distribution of the entire datasets for each modification/bound protein was visualized by box plots. Inference on comparisons was performed non-parametrically by means of a Mann-Whitney U test.

**REFERENCES**

# 1. Miccio A, Cesari R, Lotti F, Rossi C, Sanvito F, et al. (2008) In vivo selection of genetically modified erythroblastic progenitors leads to long-term correction of beta-thalassemia. Proc Natl Acad Sci U S A 105: 10547-10552.

# 2. Cattoglio C, Facchini G, Sartori D, Antonelli A, Miccio A, et al. (2007) Hot spots of retroviral integration in human CD34+ hematopoietic cells. Blood.

# 3. Cui K, Zang C, Roh TY, Schones DE, Childs RW, et al. (2009) Chromatin signatures in multipotent human hematopoietic stem cells indicate the fate of bivalent genes during differentiation. Cell Stem Cell 4: 80-93.
